# Metabolomics analysis reveals metabolite diversity of the rare cliff plant *Oresitrophe rupifraga* unge

**DOI:** 10.1016/j.heliyon.2024.e33076

**Published:** 2024-06-14

**Authors:** Hao Wang, Jinjun Cao, Sheng Chang, Caifeng Yan, Guangming Zhang

**Affiliations:** a Key Laboratory of Saline-alkali Vegetation Ecology Restoration, Ministry of Education, Northest Forestry University, Harbin, Heilongjiang 150040, China; b Department of Pharmacy, Changzhi Medical College, Changzhi, Shanxi 046000, China; c Millet Research Institute, Shanxi Agricultural University, Changzhi, Shanxi 046000, China

**Keywords:** *Oresitrophe rupifraga* bunge, Metabolites analysis, Leaves, Rhizomes, Bulblets

## Abstract

*Oresitrophe* is monotypic, with the only species, *Oresitrophe rupifraga* Bunge, which is exclusive to China, having special growth and developmental traits due to its habitat. Furthermore, it has bright flowers and medicinal benefits. This study investigated the metabolites present in various tissues of *Oresitrophe rupifraga* Bunge. Using a widely targeted metabolomics approach, 1965 different metabolites were identified in *Oresitrophe rupifraga* Bunge. Based on principal component analysis (PCA) and orthogonal partial least squares discriminant analysis (OPLS-DA), the aboveground and underground metabolites of *Oresitrophe rupifraga* differed significantly. The comparison between bulblets and leaves revealed the differential expression of 461 metabolites, whereas the comparison between rhizomes and leaves showed the differential expression of 423 metabolites, and the comparison between bulblets and rhizomes showed the differential expression of 249 metabolites. The bulblets exhibited 49 metabolites that were higher and 412 metabolites that were lower than those of the leaves, whereas the rhizomes showed 123 upregulated and 300 downregulated metabolites. Bulblets showed an increase in 18 metabolites and a decrease in 231 metabolites compared to the rhizomes. Leaves contain more phenolic acids than the rhizomes and bulblets, whereas the rhizomes and bulblets contain more terpenoids than the leaves. KEGG pathway analysis showed an association between metabolites and metabolic pathways, as well as their effect on the progression and maturation of *Oresitrophe rupifraga* Bunge. The research findings can provide some insight into the growth and developmental traits of *Oresitrophe rupifraga* Bunge, thus providing a theoretical foundation for cultivating and utilising this plant.

## Graphical abstract

**Figure:**
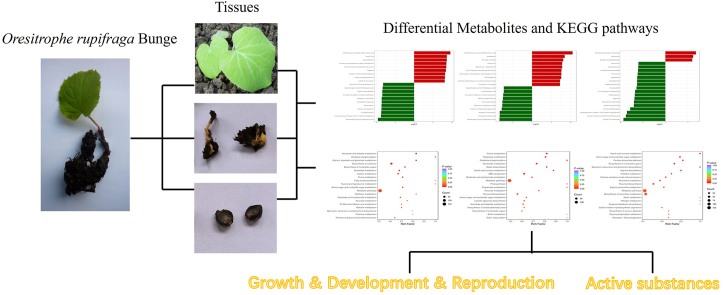

## 1 Introduction

*Oresitrophe rupifraga* Bunge is a scarce cliff-dwelling plant that typically thrives in the moist crevices of cliffs or valleys endemic to northern and central China. This perennial herb grows to a height of 12–28 cm. The leaves originate from the base of the plant and are either heart-shaped or ovate with a short, sharp apex. The edge of the leaf is serrated, its base is heart-shaped, and its petiole is 11.5–13.5 cm long [1]. The rhizome is thick and robust, with small brown bulblets. The singularity of *Oresitrophe rupifraga* Bunge is demonstrated by its various characteristics. *Oresitrophe rupifraga* Bunge, which blooms earlier in spring than many other plants, first flowers and then grows leaves (outdoors), displaying very bright flowers [2]. It can tolerate cold and dry conditions owing to its unique habitat. *Oresitrophe rupifraga* has the capacity to thrive in rock crevices, which may be attributed to the secretion of acidic substances from its roots. Erosion of rocks creates a suitable environment for plant growth. *Oresitrophe rupifraga* has a powerful capacity to absorb carbon dioxide, which can lower the amount of carbon dioxide inside buildings [3]. The *Oresitrophe rupifraga* Bunge is capable of reproduction through division propagation, with its stem discs densely populated by small bulblets. The rhizomes interconnect the small bulblets and leaves germinate from a few of the bulblets; however, multiple bulblets on the stem disc are not capable of producing leaves. Investigating the metabolic products generated by different tissues in *Oresitrophe rupifraga* Bunge can assist in understanding its development and growth processes.

Research on the chemical composition and active roles of *Oresitrophe rupifraga* Bunge has been scarce. Studies have shown that *Oresitrophe rupifraga* contains multiple chemical components that exert various effects. The entire plant has been used in traditional medicine to treat conditions linked to inflammation, such as infantile enteritis and diarrhoea [[4], [5], [6]]. Furthermore, *Oresitrophe rupifraga* Bunge has anti-neuroinflammatory activity [7]. Using widely targeted metabolomics, it is possible to reap the benefits of high throughput, heightened sensitivity, and extensive coverage [8]. Utilising a broadly targeted metabolomics method to examine the chemical components of *Oresitrophe rupifraga* Bunge can provide better insights into the distinct features of this plant.

Adversity-induced stress leads to the generation of secondary metabolites in plants and plants exhibiting higher stress resistance may produce more specific secondary metabolites. Studies have demonstrated that cephalanthine obtained from *Stephania* plants has a significant effect on reducing the COVID-19 spread [9]. Additionally, it increases the number of white blood cells and strengthens the human immune system [10]. Petroselinic fatty acids derived from Umbelliferae plants are rare with a broad range of biological effects [11]. As a flavonoid, dihydroquercetin has numerous pharmacological applications, including aiding skin repair, alleviating skin inflammation, and preventing skin cancer [12]. Cordycepin has many pharmacological effects such as antitumour, immune-regulatory, and anti-inflammatory [13]. Phytosphingosine, referred to as “plant soft gold”, is a key ingredient in the production of moisturising and skincare products [14]. Metabolomic analysis demonstrated that *Oresitrophe rupifraga* Bunge contains a range of secondary metabolites, including the aforementioned components. The objective of this study was to analyse the variety of metabolites in *Oresitrophe rupifraga* Bunge using widely targeted metabolomics. Discrepancies in metabolites between different tissues were investigated to reveal the development and growth mechanisms of *Oresitrophe rupifraga* Bunge. The results of this research will serve as a foundation for further exploration and use of this unique plant, as well as its artificial cultivation.

## 2 Materials and methods

### 2.1 Plant materials

**Fig. 1:**
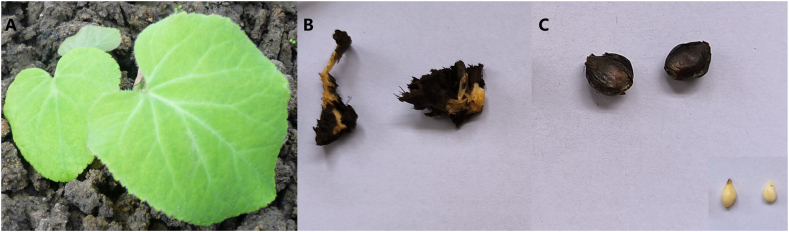
Phenotypes of different tissues of *Oresitrophe rupifraga* Bunge. (**A**) Leaves. (**B**) Rhizomes. (**C**) Bulblets (the lower right corner features the bulblets with their husk removed.).

*Oresitrophe rupifraga* Bunge plants were harvested from the wild (Wangmangling, Jincheng, Shanxi, China) and were cultivated in a greenhouse. Three tissue samples were collected from the leaves (Fig. 1A), rhizomes (Fig. 1B), and bulblets (Fig. 1C). Leaf samples were taken from the same region of different plant leaves, and rhizomes were collected from the area near the removed leaves when feasible. After collection, the samples were immediately placed in liquid nitrogen and subsequently transferred to dry ice for transportation to the testing and analysis sites. Upon arrival, it was stored in a −80 °C refrigerator until the test. Three biological replicates were used for each tissue sample. Dr. Zhang Guangming of Changzhi Medical College identified the *Oresitrophe rupifraga* Bunge plant.

### 2.2 UPLC-MS/MS analysis

Biological samples were freeze-dried under vacuum. Subsequently, the dried samples were introduced into a lyophiliser (Scientz-100F) and further processed into a powder by grinding for 1.5 min at 30 Hz using a grinder (MM 400, Retsch). Subsequently, an electronic balance (MS105DM) was used to weigh 50 mg of the ground sample. Subsequently, 1200 μL of a 70 % methanolic aqueous internal standard extract, pre-cooled at −20 °C, was introduced to the sample, ensuring that it did not exceed 50 mg. The addition rate was set at 1200 μL of extractant per 50 mg of the sample. The mixture underwent vortexing for 30 s once half an hour, and this process was repeated a total of 6 times. After centrifuging at 12,000 rpm for 3 min, the supernatant was carefully extracted. Next, the sample underwent filtration through a 0.22-μm microporous membrane. The filtered sample was subsequently transferred to an injection vial, prepared for UPLC-MS/MS analysis.

We analysed the sample extracts using a UPLC-ESI-MS/MS system, namely, the ExionLC™ AD system manufactured by Sciex (https://sciex.com.cn/). This system was used in conjunction with a tandem mass spectrometry system, which is also available from Sciex. The UPLC's conditions were set below: The column utilised was an Agilent SB-C18 with specifications of a 1.8 μm particle size and dimensions of 2.1 mm inner diameter by 100 mm length. The mobile phase consisted of solvents A (pure water with 0.1 % formic acid) and B (acetonitrile with 0.1 % formic acid). The sample was measured using a gradient program that commenced with the initial conditions of 95 % A and 5 % B. Linear gradient was programmed over a duration of 9 min, transitioning to a composition of 5 % A and 95 % B. The mixture was incubated for 1 min. Subsequently, the composition was changed to 95 % A and 5.0 % B within a time frame of 1.1 min and held at this ratio for 2.9 min. The flow rate was established at 0.35 mL/min, the column oven temperature was held at 40 °C, and the injection amount amounted to 2 μL. Subsequently, the effluent was directed into an ESI-triple quadrupole linear ion trap (QTRAP).

The ESI source's operational parameters were listed below: the source maintained a temperature of 500 °C, the ion spray voltage was 5500 V in positive ion mode and −4500 V in negative ion mode, ion source gases I and II were adjusted to 50 and 60 psi, while the curtain gas (CUR) was set at 25 psi. Additionally, collision-activated dissociation was configured at a high setting. A medium collision gas (nitrogen) was used to obtain the QQQ scans in the MRM experiments. For each specific MRM transition, both the collision energy and the declustering potential were fine-tuned for optimisation. Throughout each dedicated monitoring interval, MRM transition sets were utilised and customised for the metabolites eluted at that time.

### 2.3 Data analysis

We utilised the “prcomp” function to perform unsupervised principal component analysis (PCA) in the R programming environment. Before performing the unsupervised PCA, the data were subjected to scaling of the unit variance. The outcomes of the hierarchical cluster analysis (HCA) for the samples and metabolites are presented in heatmaps accompanied by dendrograms. We executed the HCA using the R package “ComplexHeatmap”, and the normalised metabolites' signal intensities were subjected to scaling of unit variance and depicted using colour spectra.

In the two-group analysis, the identification of differential metabolites (DMs) relied on two specific criteria: a variable importance in projection (VIP) score greater than one and an absolute Log_2_ fold change (|Log_2_FC|) equal to or exceeding 1.0. VIP values were drawn from the outcomes of the orthogonal partial least squares discriminant analysis (OPLS-DA), encompassing permutation and score plots. R package “MetaboAnalystR” was employed for analyses. The data were subjected to logarithmic transformation (log_2_) and mean centring before OPLS-DA was applied. To reduce the likelihood of over-fitting, 200 permutations were used.

KEGG (Kyoto Encyclopaedia of Genes and Genomes) is a database that merges genomic, chemical, and systemic functional information. The identified metabolites were annotated using the Kyoto Encyclopaedia of Genes and Genomes compound database. The annotated metabolites were linked to the Kyoto Encyclopaedia of Genes and Genomes pathway database. Pathways that included highly regulated metabolites were subsequently analysed using Metabolite Set Enrichment Analysis (MSEA), and the significance of these pathways was assessed using p-values calculated based on hypergeometric tests.

## 3 Results and discussion

### 3.1 Metabolite identification

**Fig. 2:**
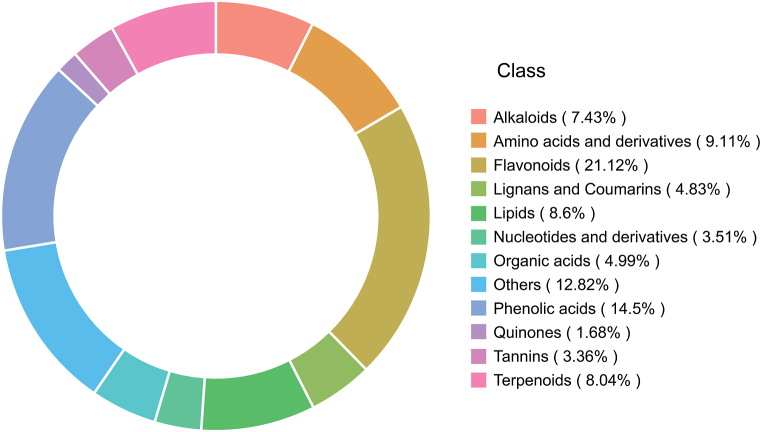
Ring diagram of identified metabolite species in *Oresitrophe rupifraga* Bunge.

We used Analyst 1.6.3 software for mass spectrometry data analysis. Broadly targeted metabolomics method was utilised to analyse metabolites qualitatively and quantitatively in the samples, with the help of the MetWare internal database and the UPLC-MS/MS system. We discovered that 1965 metabolites were present in *Oresitrophe rupifraga* Bunge. Metabolites were classified into 12 major groups. Flavonoids were the most abundant, making for 21.12 %. The types of compounds left were phenolic acids (14.5 %), amino acids and derivatives (9.11 %), lipids (8.6 %), terpenoids (8.04 %), alkaloids (7.43 %), organic acids (4.99 %), lignans and coumarins (4.83 %), nucleotides and derivatives (3.51 %), tannins (3.36 %), quinones (1.68 %). Additionally, 12.82 % of the compounds belonged to other classes (Fig. 2). The number of metabolites identified in *Oresitrophe rupifraga* exceeded those in previous related studies. Studies have been conducted on fruits [[15], [16], [17]], leaves [18,19], roots [20,21], and bulbs [22] of plants. In these studies, the number of identified metabolites varied from 403 to 1088. *Oresitrophe rupifraga* Bunge is an ideal resource for natural product research and development because of its distinct features.

### 3.2 Multivariate analysis of metabolites in Oresitrophe rupifraga Bunge

**Fig. 3:**
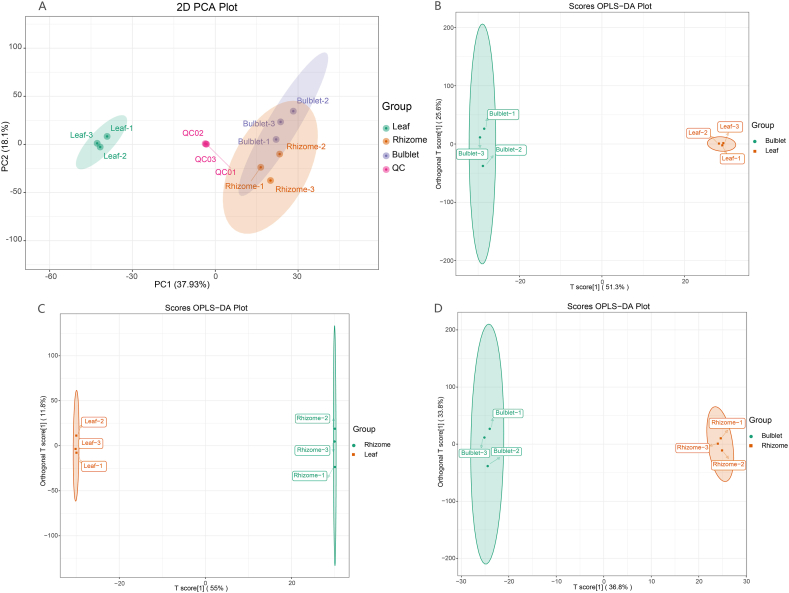
Multivariate analysis of metabolites identified. (A) PCA analysis of metabolites identified from *Oresitrophe rupifraga* Bunge. Quality control (QC) samples are formulated by blending experimental sample extracts. (B–D) OPLS-DA of metabolites identified among bulblets, rhizomes, and leaves.

PCA can be applied to the metabolites found in samples, including quality control samples. The PCA results classified samples from the three different tissues and quality control (QC) samples into four groups. The QC quality control samples showed a high level of aggregation, indicating good stability. PC1 and PC2 clearly differentiated the three sets of experimental samples, with PC1 separating the aboveground (leaf) and underground (rhizome and bulblet) parts, and PC2 distinguishing the two underground sets (Fig. 3A). The cluster heatmap is analogous to this (Fig. S1). The variance between the aboveground and underground samples was substantial, whereas the discrepancy between the two underground samples was minor. Leaf tissue samples showed the highest degree of intragroup aggregation, followed by rhizome samples, and bulblet samples were the least aggregated. The tight connection between the rhizomes and bulblets resulted in a higher degree of similarity in their metabolites. A large quantity of metabolites in plants may affect the principal component analysis. The developmental features of each bulblet varied greatly (certain bulblets were able to generate foliage, whereas others were not) and could be closely related to metabolites. To delve deeper into the differences in metabolite composition between each pair of sample groups, OPLS-DA models were employed to assess the distinctive metabolite characteristics of the two groups under consideration. The detailed prediction parameters were R^2^X, R^2^Y, and Q^2^, and the results of these parameters between the bulblet and leaf (R^2^X = 0.769, R^2^Y = 1, Q^2^ = 0.941), rhizome and leaf (R^2^X = 0.669, R^2^Y = 1, Q^2^ = 0.953), and bulblet and rhizome (R^2^X = 0.795, R^2^Y = 1, Q^2^ = 0.803) were reliable (when Q^2^ and R^2^Y approached 1, the model became increasingly stable and dependable) (Fig. S2). In these models, each pair of sample groups was distinctly separated, as illustrated in Fig. 3B–D. This separation indicated significant differences in the metabolic characteristics between each pair of sample groups.

### 3.3 Differential metabolite analysis

**Fig. 4:**
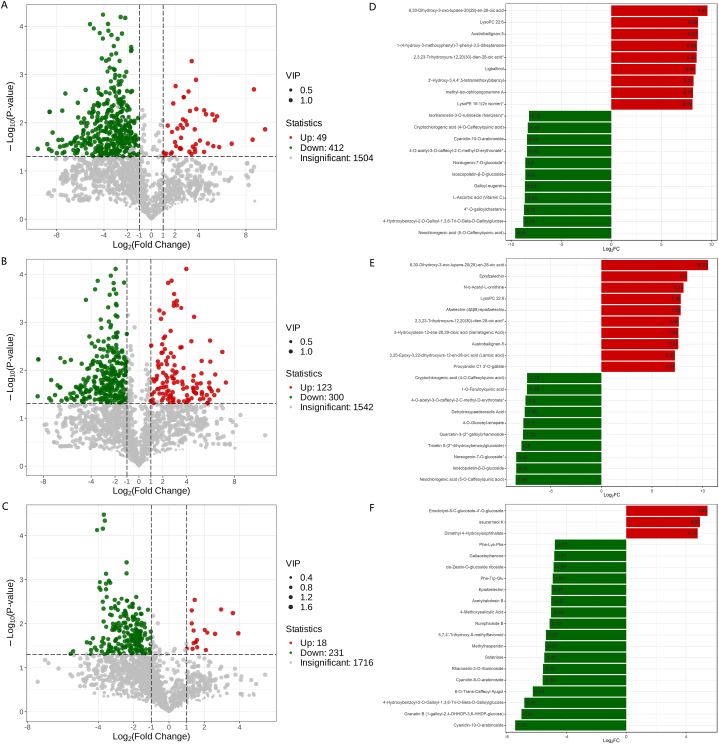
Metabolites of *Oresitrophe rupifraga* Bunge vary between different tissues. (A)–(C) Volcano plot of DMs identified. DMs were metabolites with VIP >1, FC ≥ 2 or ≤0.5, and p-value ≤0.05 in each two tissues. (D)–(F) Top 20 most significant DMs according to FC values. (A) Bulblet vs. leaf. (B) Rhizome vs. leaf. (C) Bulblet vs. rhizome. (D) Bulblet vs. leaf. (E) Rhizome vs. leaf. (F) Bulblet vs. rhizome.

Metabolomic data are characterised by their high dimensionality and extensive nature. Therefore, it was crucial to employ a combination of univariate and multivariate statistical analyses. This comprehensive approach allows for a thorough examination of data from various perspectives, ultimately leading to the precise identification of DMs. In this study, the criteria used for screening DMs were derived from the OPLS-DA model's results, VIP >1, and FC ≥ 2 or FC ≤ 0.5. Following the screening process, 1003 metabolites were differentially expressed between the bulblets and leaves, 992 between the rhizomes and leaves, and 788 between the bulblets and rhizomes. Added p-value ≤0.05 as the screening criterion, 461 metabolites were found to be differentially expressed between bulblets and leaves, 423 between rhizomes and leaves, and 249 between bulblets and rhizomes (Fig. 4A–C). When compared to the leaves, 49 metabolites were found to be upregulated and 412 were downregulated in the bulblets (Fig. 4A), whereas 123 metabolites were upregulated and 300 were downregulated in the rhizomes (Fig. 4B). The bulblets showed an increase in 18 metabolites and a decrease in 231 metabolites compared to the rhizomes (Fig. 4C). The upregulation of metabolites in the leaves was much greater than that in the bulblets and rhizomes. Of the top 20 most significantly differentially expressed metabolites (VIP >1, FC ≥ 2 or ≤ 0.5), the bulblets had an elevated expression of nine metabolites and a reduced expression of eleven metabolites in comparison with the leaves, the rhizomes had a growing expression of ten metabolites and a diminishing expression of ten metabolites in comparison with the leaves, the bulblets had a heightened expression of three metabolites and a declining expression of seventeen metabolites in comparison with the rhizomes (Fig. 4D–F). In comparison to the leaves, the bulblets had a maximum upregulated metabolite fold change of 785.37, a maximum downregulated metabolite fold change of 779.42, and an average fold change of 395.85, 411.72, respectively. In comparison to the leaves, the rhizomes had a maximum upregulated metabolite fold-change of 1506.94, a maximum downregulated metabolite fold-change of 356.00, and average fold-changes of 351.53, 240.41, respectively. In comparison to rhizomes, bulblets had a maximum upregulated metabolite fold-change of 43.30, a maximum downregulated metabolite fold-change of 172.32, and an average fold-change of 33.78, 56.50. This reiterated that there was a marked difference in metabolites between the aboveground and underground parts of *Oresitrophe rupifraga* Bunge, although distinctions between different tissues in the underground portion were comparatively minor. The results suggest that there was a greater content of many metabolites in the leaves than in the rhizomes and bulblets; however, there were some unique metabolites that were significantly more abundant in the rhizomes and bulblets than in the leaves. Most of the metabolites found in the rhizomes were more abundant than those in the bulblets.

Among the top few downregulated metabolites in the comparison of bulblets and leaves, neochlorogenic acid (5-*O*-caffeoylquinic acid) was the top 1 metabolite (FC value equivalent to 779.42-fold), whereas the comparison between rhizomes and leaves was identical (FC value equivalent to 356.00-fold). These results are similar to those reported by Li et al. [5]. Various plants contain chlorogenic acids, which belong to the class of phenolic compounds [[23], [24], [25]]. In plants, caffeoylquinic acid is present in three forms in plants: 3-*O*-caffeoylquinic acid, 5-*O*-caffeioylquinic acid, and 4-*O*-caffeoylquinic acid [26]. 5-*O*-caffeoylquinic acid has been found to possess antioxidant, anti-inflammatory, neuroprotective, anticancer, antidiabetic, cardiovascular protective, and antiviral activities [[27], [28], [29], [30], [31], [32], [33]]. Noreugenin-7-*O*-glucoside, isoscopoletin-β-D-glucoside, 4-*O*-acetyl-3-*O*-caffeoyl-2-C-methyl-d-erythronate, cryptochlorogenic acid (4-*O*-caffeoylquinic acid) were also among the top few downregulated metabolites in both the comparison between bulblets and leaves, as well as in the comparison between rhizomes and leaves. Among the top few upregulated metabolites in the comparison between bulblets and leaves, 6,30-Dihydroxy-3-oxo-lupane-20(29)-en-28-oic acid was the top 1 metabolite (785.37-fold), whereas the comparison between rhizomes and leaves was identical (1506.94-fold). This compound, similar to lupinane-type pentacyclic triterpenoids, is likely to exhibit a range of biological activities [34,35]. Epiafzelechin, a compound that was produced in greater content in rhizomes than in leaves (361.80-fold), has the potential to be used as a medication for diabetes [36]. 2,3,23-Trihydroxyurs-12,20(30)-dien-28-oic acid and LysoPC 22:6 were among the top few upregulated metabolites in the comparison between bulblets and leaves, as well as in the comparison between rhizomes and leaves.

Out of 461 differentially expressed metabolites between leaves and bulblets (VIP >1, FC ≥ 2 or ≤ 0.5, p-value ≤0.05), 68 phenolic acid metabolites were upregulated, while only 2 were downregulated. The proportion of the two patients was 34:1. The relationship between the rhizomes and bulblets was comparable, with a 40:1 ratio of upregulated to downregulated phenolic acids. Leaves and rhizomes exhibited a 2.9:1 ratio of upregulated to downregulated phenolic acids. The aboveground parts of the plants exhibited a higher concentration of phenolic acids in terms of both type and content than the underground parts, with the rhizomes having an intermediate state of phenolic acids among the leaves, rhizomes, and bulblets. The proportions of metabolites that were upregulated and those that were downregulated by the other types of rhizomes and leaves were all in an intermediate state (Table 1). The data in Table 1 indicate that the underground tissues contain a greater amount of terpenoids than the aboveground parts, and the rhizomes contain more tannins than the leaves. Leaves contain a much larger quantity of amino acids and derivatives and nucleotides and derivatives than bulblets. Leaves contained more amino acids and derivatives than rhizomes, with a marginal increase in nucleotides and derivatives. Rhizomes contained a greater abundance of nucleotides and derivatives (DMs contained only upregulated nucleotides and derivatives) than bulblets, with a marginal increase in amino acids and derivatives. The metabolites suggested that aboveground life activities were quite active, with rhizomes being more vigorous than small bulblets. Subsequent Kyoto Encyclopaedia of Genes and Genomes functional annotation and enrichment analysis of the DMs verified this finding. Nucleotides and their derivatives could potentially be the deciding factors for bulblet sprouting and leaf growth. Most bulblets remained dormant for extended durations. This approach could be combined with transcriptomic research to further investigate the underlying mechanisms. For instance, we collected samples of both dormant and sprouting bulbs under identical conditions for transcriptome sequencing to analyse the variations in gene expression levels between the two states and to link the differentially expressed genes with metabolites.

**Table 1:** Comparison of upregulation and downregulation of various DMs between different tissues.

Tissues	Metabolite types	Upregulated metabolites: downregulated metabolites
L vs. B	Phenolic acids	34:1
	Nucleotides and derivatives	22:1
	Amino acids and derivatives	19.3:1
	Flavonoids	18:1
	Organic acids	16:1
	Lignans and Coumarins	15.5:1
	Alkaloids	9.7:1
	Lipids	5.4:1
	Terpenoids	0.7:1
L vs. R	Amino acids and derivatives	5.8:1
	Lipids	5.1:1
	Organic acids	5:1
	Lignans and Coumarins	3.4:1
	Phenolic acids	2.9:1
	Nucleotides and derivatives	2.1:1
	Alkaloids	2:1
	Flavonoids	1:1
	Tannins	0.9:1
	Terpenoids	0.8:1
R vs. B	Nucleotides and derivatives	15:0
	Phenolic acids	40:1
	Flavonoids	14.7:1
	Tannins	14:1
	Amino acids and derivatives	10.5:1
	Lignans and Coumarins	9.5:1
	Terpenoids	2.5:1
	Lipids	0.5:1

(L: leaf; R: rhizome; B: bulblet).

### 3.4 Differential metabolites' KEGG functional annotation and enrichment analysis

**Fig. 5:**
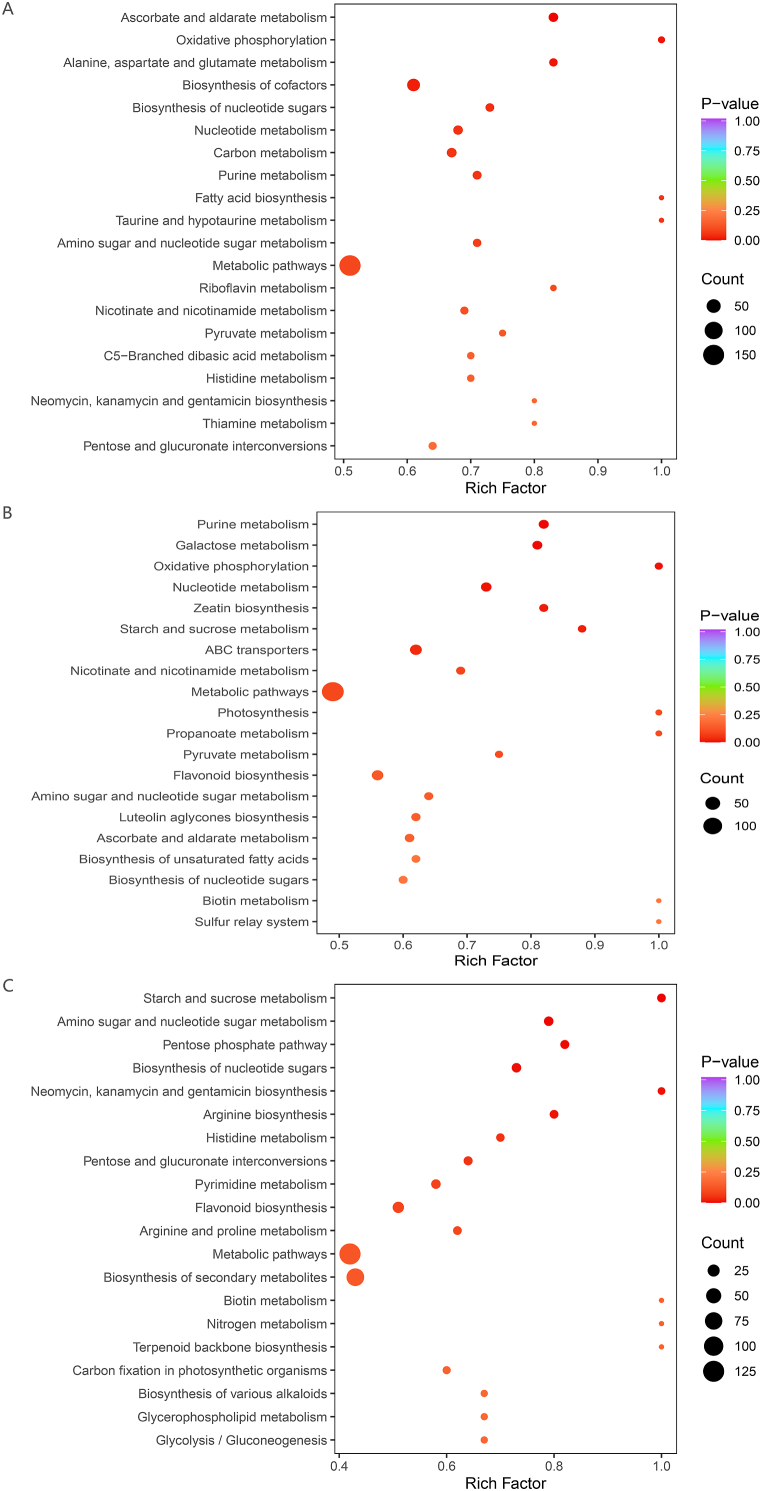
Rich factor values for annotated differentially expressed metabolites within the KEGG database. (A) Bulblet vs. leaf. (B) Rhizome vs. leaf. (C) Bulblet vs. rhizome.

To establish connections between metabolites and metabolic pathway networks, we categorised the differentially expressed metabolites into pairs and linked them to the KEGG database. Among the differentially expressed metabolites observed in the bulblets and leaves, 154 (69.06 %) were annotated as part of metabolic pathways, 84 (37.67 %) were linked to the biosynthesis of secondary metabolites, and 38 (17.04 %) were associated with cofactor biosynthesis. Of the differentially expressed metabolites in the rhizomes and leaves, 148 (69.16 %) were annotated as part of metabolic pathways, 83 (38.79 %) were linked to the biosynthesis of secondary metabolites, and 31 (14.49 %) were connected to the biosynthesis of cofactors. A total of 126 (69.23 %) metabolites that were differentially expressed in bulblets and rhizomes were involved in metabolic pathways, whereas 78 (42.86 %) and 27 (14.84 %) metabolites were linked to the biosynthesis of secondary metabolites and cofactors, respectively (Fig. S3). Next, KEGG pathway enrichment analysis was performed to analyse the disparities within the metabolic pathways of each group. For bulblets and leaves, the pathways with the most notable enrichment were ascorbate and aldarate metabolism (ko00053); oxidative phosphorylation (ko00190); alanine, aspartate, and glutamate metabolism (ko00250); cofactor biosynthesis (ko01240); and biosynthesis of nucleotide sugars (ko01250) (arranged by p-value) (Fig. 5A). For rhizomes and leaves, the pathways with the highest degree of enrichment were purine metabolism (ko00230), galactose metabolism (ko00052), oxidative phosphorylation (ko00190), nucleotide metabolism (ko01232), zeatin biosynthesis (ko00908) (Fig. 5B). For bulblets and rhizomes, the pathways with the highest degree of enrichment were metabolism of starch and sucrose (ko00500), metabolism of amino and nucleotide sugars (ko00520), pentose phosphate (ko00030), biosynthesis of nucleotide sugars (ko01250), and neomycin, kanamycin, and gentamicin biosynthesis (ko00524) (Fig. 5C).

**Fig. 6:**
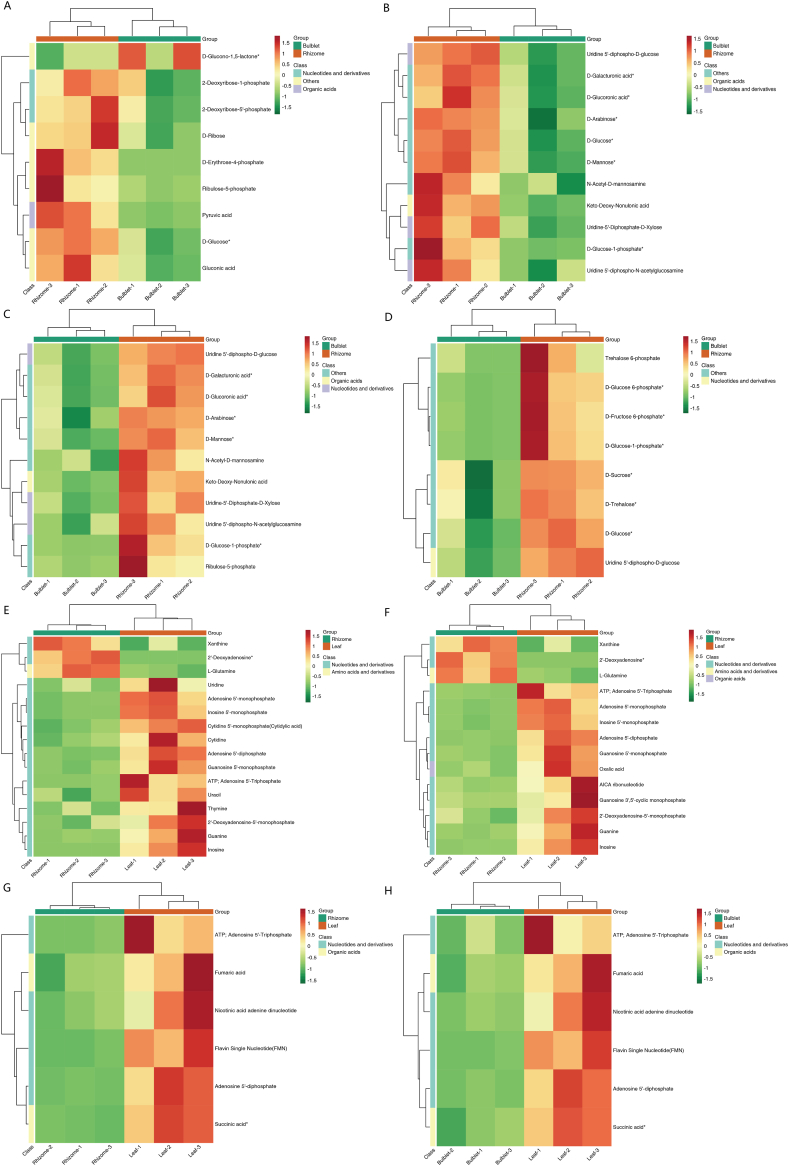
Cluster heatmap of DMs in KEGG pathways. (A) Pentose phosphate pathway, Bulblet vs. rhizome. (B) Amino sugar and nucleotide sugar metabolism, Bulblet vs. rhizome. (C) Biosynthesis of nucleotide sugars, Bulblet vs. rhizome. (D) Starch and sucrose metabolism, Bulblet vs. rhizome. (E) Nucleotide metabolism, Rhizome vs. leaf. (F) Purine metabolism, Rhizome vs. leaf. (G) Oxidative phosphorylation, Rhizome vs. leaf. (H) Oxidative phosphorylation, Bulblet vs. leaf.

The significant metabolic pathway differential metabolite heatmap clearly demonstrates the results of the previous discussion. In the pentose phosphate pathway, metabolism of amino sugars and nucleotide sugars, nucleotide sugar biosynthesis, and metabolism of starch and sucrose, almost all metabolites that were differentially expressed in rhizomes were increased compared to those in bulblets (Fig. 6A–D). This may explain the increased number of nucleotides and derivatives in the rhizomes. The pentose phosphate pathway is an important metabolic pathway in plants. It serves as the principal source of reduced nicotinamide adenine dinucleotide phosphate (NADPH) and is responsible for the generation of ribulose 5-phosphate, ribose 5-phosphate, and erythrose 5-phosphate. These compounds play essential roles in the synthesis of nucleotides, aromatic amino acids, and fatty acids, particularly in non-photosynthetic tissues [37]. Research has indicated that GA3-induced dormancy release in *Leymus chinensis* seeds is strongly correlated with the metabolism of starch and sucrose, as well as the metabolism of amino sugars and nucleotide sugars [38]. In the oxidative phosphorylation metabolic pathway, all metabolites that were differentially expressed in leaves were more abundant than those in the rhizomes and bulblets (Fig. 6G–H). Oxidative phosphorylation is an essential metabolic pathway through which organisms obtain energy. This implied that the life activities of the aboveground parts were the most energetic (increased oxidative phosphorylation metabolic activity). Regarding nucleotide and purine metabolism, rhizomes had fewer upregulated differential metabolites than leaves (three differential metabolites were more abundant than in leaves) (Fig. 6E–F), suggesting that their life activities were in a moderate state.

## 4 Conclusions

This study used a widely targeted metabolomics method to analyse the metabolites in the leaves, rhizomes, and bulblets of *Oresitrophe rupifraga* Bunge. In total, 1965 metabolites have been discovered in various tissues of *Oresitrophe rupifraga* Bunge, many of which possess diverse biological activities. Metabolites varied significantly between different tissues and could be categorised into two groups: aboveground and underground. The leaves of the plant (aboveground part) contained greater quantities of phenolic acids, amino acids and derivatives, nucleotides and derivatives. Terpenoids are much more abundant in the rhizomes and bulblets (underground parts) than in the leaves. Compared to the leaves, the rhizomes featured a greater amount of tannins. Variations in metabolites between different tissues might be associated with the growth and development of *Oresitrophe rupifraga* Bunge. The analysis conducted in this study investigated the types and concentrations of metabolites found in different tissues of *Oresitrophe rupifraga* Bunge, revealing specific patterns in the differences in metabolites between these tissues. However, research on the growth, development, and environmental adaptation mechanisms of *Oresitrophe rupifraga* remains lacking. The application of proteomics research enables us to enhance our understanding of the environmental signal response and adaptation mechanisms of *Oresitrophe rupifraga*, the signal transduction of plant hormones, and protein discrepancies in different tissues and organs. The living environment of *Oresitrophe rupifraga* Bunge is unique, and a detailed study is required to reveal its unique mechanisms. Once the assembly of a high-quality genome map for *Oresitrophe rupifraga* Bunge is completed, it will greatly enhance future studies on the biological functions of genes. Further research is required to gain deeper insights into the association between metabolites and the growth and development of *Oresitrophe rupifraga* Bunge.

## Data availability statement

Has data associated with your study been deposited into a publicly available repository?

No, Data will be made available on request.

## Ethics statements

There were no human subjects and mammalian animal experiments in our research.

## CRediT authorship contribution statement

**Hao Wang:** Writing – original draft, Resources, Methodology, Investigation, Funding acquisition, Formal analysis, Data curation. **Jinjun Cao:** Resources. **Sheng Chang:** Methodology. **Caifeng Yan:** Writing – review & editing. **Guangming Zhang:** Writing – review & editing, Supervision, Conceptualization.

## Declaration of competing interest

The authors declare that they have no known competing financial interests or personal relationships that could have appeared to influence the work reported in this paper.
