# Supplementary material for: Metabolomics analysis reveals metabolite diversity of the rare cliff plant Oresitrophe rupifraga unge
Source: Heliyon. 2024 Jun 14;10(12):e33076. doi: 10.1016/j.heliyon.2024.e33076 (PMC11211885; doi:10.1016/j.heliyon.2024.e33076)
Supplement: Multimedia component 1 [file mmc1.docx]

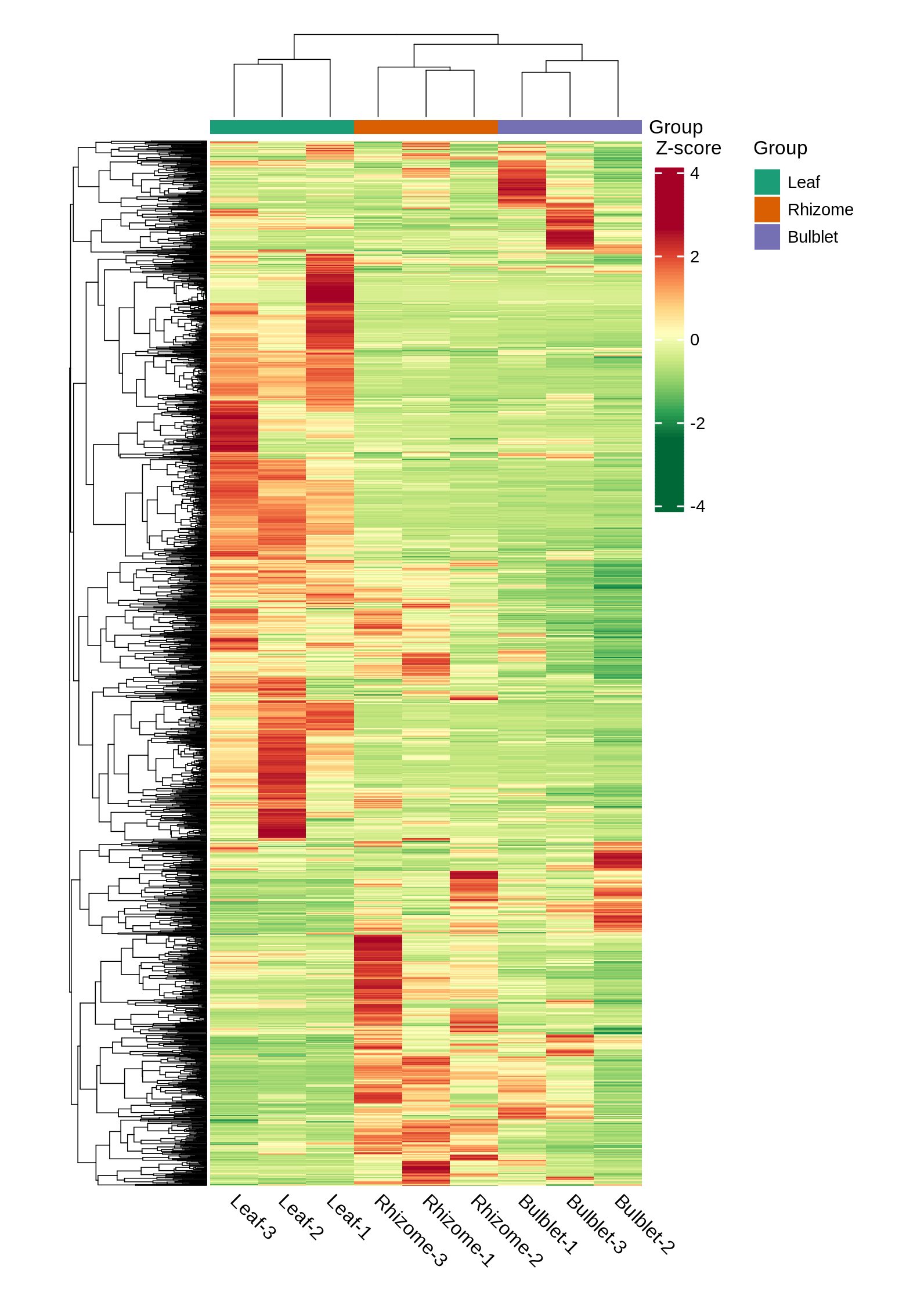


**Figure S1.** Sample population clustering heatmap. The cluster line on the left side of the figure is the metabolite cluster line, and the cluster line above the figure is the sample cluster line.


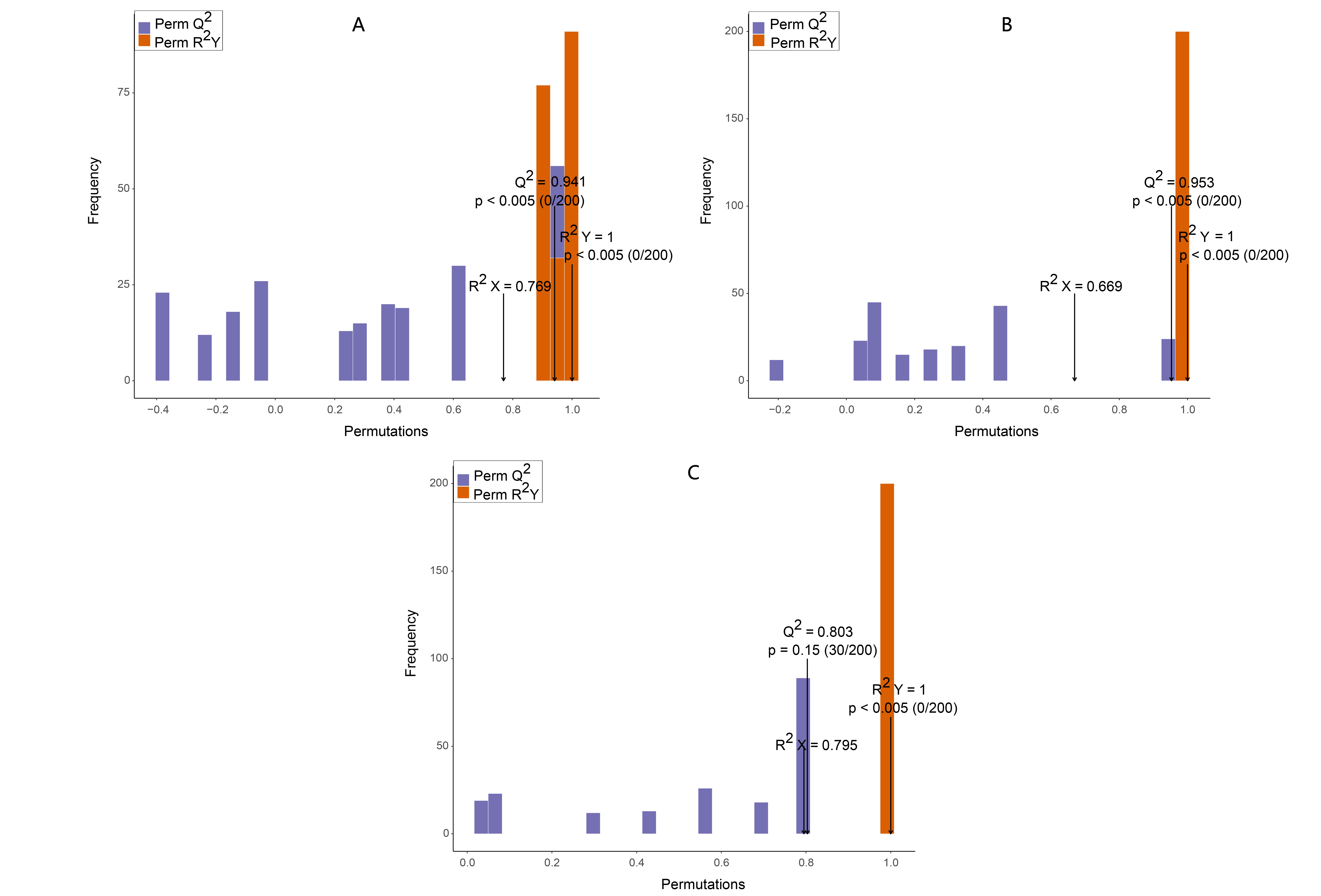


**Figure S2.** OPLS-DA verification map. (A) Bullet vs. leaf. (B) Rhizome vs. leaf. (C) Bulblet vs. rhizome.

Note: Abscissa represents the values of model R^2^Y and Q^2^, and ordinate is the frequency of model classification effect in 200 random permutation and combination experiments. In the figure, the orange represents the random grouping model R^2^Y, the purple represents the random grouping model Q^2^, and the black arrow represents the R^2^X, R^2^Y and Q^2^ values of the original model.





**Figure S3.** Summary of the different functions annotated for the differentially expressed metabolites (DEMs) using the KEGG database. (A) Bullet vs. leaf. (B) Rhizome vs. leaf. (C) Bulblet vs. rhizome.
